# CXCR3 Provides a Competitive Advantage for Retention of *Mycobacterium tuberculosis*-Specific Tissue-Resident Memory T Cells Following a Mucosal Tuberculosis Vaccine

**DOI:** 10.3390/vaccines11101549

**Published:** 2023-09-29

**Authors:** Ellis Armitage, Diana Quan, Manuela Flórido, Umaimainthan Palendira, James A. Triccas, Warwick J. Britton

**Affiliations:** 1 Centenary Institute, The University of Sydney, Sydney, NSW 2006, Australia; ellisaway@hotmail.com (E.A.); d.quan@centenary.org.au (D.Q.); m.florido@centenary.org.au (M.F.); umaimainthan.palendira@sydney.edu.au (U.P.); 2 School of Medical Sciences, Faculty of Medicine and Health, The University of Sydney, Sydney, NSW 2006, Australia; jamie.triccas@sydney.edu.au; 3 The University of Sydney Infectious Diseases Institute (Sydney ID), Faculty of Medicine and Health, The University of Sydney, Sydney, NSW 2006, Australia; 4 Central Clinical School, Faculty of Medicine and Health, The University of Sydney, Sydney, NSW 2006, Australia; 5 Department of Clinical Immunology, Royal Prince Alfred Hospital, Camperdown, NSW 2050, Australia

**Keywords:** CXCR3, pulmonary vaccine, tuberculosis, influenza, mouse, CD4^+^ T cell, resident memory T cell

## Abstract

*Mycobacterium tuberculosis* is a major human pathogen, and new vaccines are needed to prevent transmission. Mucosal vaccination may confer protection against *M. tuberculosis* by stimulating tissue-resident memory (T_RM_) CD4^+^ T cells in the lungs. The chemokine receptor CXCR3 promotes lung recruitment of T cells, but its role in T_RM_ development is unknown. This study demonstrates the recombinant influenza A virus vaccine PR8.p25, expressing the immunodominant *M. tuberculosis* T cell epitope p25, induces CXCR3 expression on p25-specific CD4^+^ T cells in the lungs so that the majority of vaccine-induced CD4^+^ T_RM_ expresses CXCR3 at 6 weeks. However, CXCR3^−/−^ mice developed equivalent antigen-specific CD4^+^ T cell responses to wild-type (WT) mice following PR8.p25, and surprisingly retained more p25-specific CD4^+^ T_RM_ in the lungs than WT mice at 6 weeks. The adoptive transfer of CXCR3^−/−^ and WT P25 T cells into WT mice revealed that the initial recruitment of vaccine-induced CD4^+^ T cells into the lungs was independent of CXCR3, but by 6 weeks, CXCR3-deficient P25 T cells, and especially CXCR3^−/−^ T_RM_, were significantly reduced compared to CXCR3-sufficient P25 T cells. Therefore, although CXCR3 was not essential for CD4^+^ T_RM_ recruitment or retention, it provided a competitive advantage for the induction of *M. tuberculosis*-specific CD4^+^ T_RM_ in the lungs following pulmonary immunization.

## 1. Introduction

Tuberculosis (TB) is among the top ten causes of human death [1]. The only approved vaccine, *Mycobacterium bovis* Bacillus Calmette–Guérin (BCG), is effective against childhood TB but provides weak protection in adults [2,3]. CD4^+^ T cells and IFN-γ are essential for immunity to *M. tuberculosis* [4,5,6], but vaccines that induce potent anti-*M. tuberculosis* IFNγ^+^CD4^+^ T cell responses have failed to protect humans from the disease [7]. As such, new vaccination approaches are urgently needed to prevent pulmonary TB. Novel strategies include pulmonary immunization with BCG, virus-vectored vaccines and protein subunit vaccines to stimulate memory cellular responses at the site of infection [8,9,10]. Without more effective correlates of protective efficacy, however, the efficient evaluation of these vaccine strategies proves challenging.

Tissue-resident memory cells (T_RM_) are a subset of memory T cells that permanently reside in peripheral tissues such as the lungs, in comparison to circulating effector and central memory T cells [11,12]. This positioning at the site of infection allows for them to rapidly respond to respiratory pathogens such as *M. tuberculosis*, which usually evades detection by T cells for days or weeks after initial infection [13]. Indeed, studies show that CD4^+^ and CD8^+^ T_RM_ can provide protective immunity against viruses, parasites, bacteria and fungi [14,15,16,17,18,19]. The delivery of TB vaccines to the lung also stimulates antigen-specific T_RM_ that protect against *M. tuberculosis* [20,21]. For example, the pulmonary vaccination of mice with a recombinant Influenza A virus (rIAV) expressing the *M. tuberculosis* CD4^+^ T cell epitope, Ag85B_240–254_ (PR8.p25), generated p25-specific CD4^+^ T_RM_ in the lungs that conferred protection against *M. tuberculosis* infection independent of circulating memory T cells [9]. Furthermore, the pulmonary delivery of BCG, non-tuberculous mycobacteria and protein TB vaccines also induce CD4^+^ and CD8^+^ TRM in the lungs that contributes to protection against *M. tuberculosis* infection [20,21,22]. The mechanisms that promote the development and retention of T_RM_ remain poorly understood, hindering the rational design of T_RM_-inducing vaccines. The expression of chemokine receptors regulates the recruitment of T cells to sites of infection [23]; for example, CXCR6 is important for the positioning of CD8^+^ T_RM_ in the lung airways [24]. Here, we have investigated the role of the chemokine receptor, CXCR3, in the T_RM_ response to pulmonary immunization.

CXCR3 is mainly expressed by T cells, in addition to macrophages [25], NK cells [26] and other leucocytes. CXCR3 contributes to T cell homing to many tissues, including the lung during viral infection [27,28], and it promotes leukocyte migration in response to the IFN-γ-inducible ligands CXCL9, 10 and 11. CXCL10 is upregulated in the human lung during active TB [29], recruiting CD4^+^ T cells to the lungs via CXCR3 during *M. tuberculosis* infection [30]. CXCR3 is expressed in a population of CD4^+^ T cells that are protective against *M. tuberculosis* infection in mice [31]. In addition, the CXCR3-mediated positioning of CD4^+^ T cells in the lymph nodes of mice optimizes T cell-Dendritic Cell (DC) interactions to facilitate T cell activation, another necessary step in the CD4^+^ T cell response to vaccines [32]. During *M. tuberculosis* infection in humans, CXCR3^+^ CD4^+^ T cells accumulate within the lung parenchyma and may contribute to containing bacterial growth [33]. Therefore, CXCR3 may also play a role in the vaccine-induced recruitment of CD4^+^ T cells and their retention in the lungs.

We hypothesized that CXCR3 was necessary for CD4^+^ T cells to enter the lungs and differentiate into T_RM_ in response to mucosal immunization with the rIAV vaccine, PR8.p25 [34]. In this study, we demonstrate that CXCR3 was expressed in antigen-specific CD4^+^ T cells that were recruited to the lungs in response to pulmonary immunization with a PR8.p25. Although CXCR3 was not essential for the development of antigen-specific T_RM_ in the lungs, the expression of CXCR3 on vaccine-induced CD4^+^ T cells provided a competitive advantage for their development and retention in the lungs as T_RM_.

## 2. Materials and Methods

### 2.1. Mouse Strains

All mice had a C57Bl/6 (B6) background. Female WT mice were purchased from Australian BioResources (Moss Vale, NSW, Australia). B6.129P2-Cxcr3^tm1Dgen^/J (CXCR3^−/−^) mice and B6.129S4-Cxcr3^tm1Arsa^/SoghJ (CiBER) mice were purchased from The Jackson Laboratory (Bar Harbour, ME, USA). Mice expressing transgenic T cell receptors specific for p25 (P25 mice) were kindly provided by Professor Joel Ernst (University of California, San Francisco, CA, USA). P25 mice were crossed with CXCR3^−/−^ mice in-house. All experiments involving mice were approved by the Sydney Local Health District Animal Welfare Committee under protocols 2013-075B and 2016-044. The animals were housed in the Centenary Institute Animal Facility and maintained under specific pathogen-free conditions.

### 2.2. rIAV Vaccine and Immunization

The recombinant H1N1 PR8.p25 virus was engineered to express the p25 peptide (FQDAYNAAGGHNAVF) from *M. tuberculosis* Antigen 85B_240–255_ (Ag85B, Rv1886c) as previously described [34,35]. This peptide contains an immunodominant, IA^b^-restricted CD4^+^ T epitope recognised by P25 T cell receptor transgenic mice [36,37]. In brief, cDNA of each of the eight viral genomic segments was prepared, with the modified form of the NA gene incorporating the p25 peptide. These were transfected into a co-culture of human embryonic kidney 293 (HEK293) cells and Madin–Darby canine kidney cells (MDCK). Viruses generated from this culture were collected and used to infect embryonated chicken eggs, and the rIAV was recovered from these eggs after 48 h. Viral titre was determined by plaque assay. Recombinant NA gene expression was confirmed by amplifying viral RNA by reverse transcriptase polymerase chain reaction (RT-PCR), followed by DNA sequencing.

For immunization, mice were anaesthetised with 56 mg/kg body weight ketamine and 7 mg/kg body weight xylazine, administered by intraperitoneal injection. Mice were immunized by the intranasal (i/n) route by delivering 50 µL PBS containing 20 pfu of PR8.p25 to their nostrils. From 3 days post-immunization (p.i.), mice were monitored and weighed daily until fully recovered.

### 2.3. Preparation of Single-Cell Suspensions

Following sacrifice, the lungs were perfused through the left ventricle of the heart with 10 mL PBS, except when intravascular staining (IVS) was performed to distinguish leukocytes in the lung parenchyma from those in the vasculature [38]. For IVS, mice were injected i.v. with 1 µg anti-CD45-APC-Cy7 antibody in 200 µL PBS 3–5 min prior to euthanasia. Lungs were diced and incubated in 5 mL Roswell Park Memorial Institute media containing 10% fetal calf serum (cRPMI) with 50 U/mL collagenase type IV (Sigma) and 26 µg/mL DNAse type I (Sigma) for 30 min at 37 °C. Red blood cells were lysed in ACK lysis buffer and cells resuspended in cRPMI.

### 2.4. Antibodies

The antibodies and staining reagents used for cell staining in flow cytometry and for binding IFN-γ in enzyme-linked immunospot (ELISpot) and experiments are listed in Table 1.

### 2.5. Flow Cytometry and Intracytoplasmic Cytokine Staining

For antigen stimulation and Intracytoplasmic Cytokine Staining (ICS) assays, 10^6^ cells from each suspension were cultured at 37 °C, 4 h in a U-bottom 96-well plate in 200 µL of cRPMI containing 10 µg/mL Brefeldin A and either no stimulant (negative controls), 10 µg/mL p25 peptide (GenScript; Piscataway, NJ, USA), or 50 µg/mL anti-CD3 antibody and 25 µg/mL anti-CD28 antibody (positive controls). Cells were resuspended in 40 µL of cRPMI with 1% FCS and 10 µg/mL of MHC IA^+^-p25 tetramer (NIH Tetramer Core Facility, Atlanta, GA, USA), and incubated at 37 °C for 1 h. Cells were incubated with Fc-block at 4 °C, for 20 min, then antibody staining was performed at 4 °C, 20 min. For experiments with biotinylated antibodies, cells were resuspended in 100 µL FACS buffer containing streptavidin-conjugated fluorophore (Invitrogen) at 4 °C, 20 min. For ICS, cells were then suspended in 100 µL Fix/Perm (BD) for 20 min at 4 °C. Cytokine staining was performed in 1× Perm/Wash at 4 °C, 20 min (BD). Cells were resuspended in 100 µL of formalin and run on an LSR Fortessa (BD). Gating to identify CD3^+^CD4^+^ T lymphocytes (Figure S1A–D) and cytokine^+^CD3^+^CD4^+^ T cells (Figure S2A–C) was performed using Flowjo version 9.8.2. Boolean gating was used to identify cells that expressed cytokines, singly or in combination.

### 2.6. Adoptive Transfer

For adoptive transfer experiments, splenocytes were obtained from naïve P25 RAG^−/−^ mice that were either CXCR3^wt/wt^ or CXCR3^−/−^ and CD4^+^ T cells in the suspensions quantified by flow cytometry. Then, 25,000 P25 CD4^+^ T cells of each type were transferred by i.v. into B6 mice.

### 2.7. IFNγ ELISpot

The frequency of IFN-γ-producing T cells in tissue samples was assessed by ELISpot. MultiScreen^®^ 96-well filter plates (Merck; Rahway, NJ, USA) were wetted with 20 µL of 35% ethanol and washed with PBS. Anti-IFN-γ monoclonal antibody (mAb; clone AN18), 15 µg/mL in PBS, was added to each well and incubated for 16 h at 4 °C. Wells were blocked with cRPMI (2 h, 37 °C) and washed with PBS. 0.5 × 10^5^ lung cells or 1 × 10^5^ spleen cells were added to each well in the presence of 10 µg/mL antigen (see specific experiments) or 5 µg/mL concanavalin A (Sigma; Melbourne, Australia) in cRPMI. Plates were incubated at 37 °C, 5% CO_2_ overnight. Wells were washed with PBS containing 0.01% Tween-20. Anti-IFN-γ-biotin mAb (clone XMG-1.2), 2.5 µg/mL in PBS, was added and incubated for 2 h at 37 °C. Then, the wells were washed as before and 1:1000 avidin–alkaline phosphatase (Sigma) in PBS added to each well and incubated for 45 min at room temperature, followed by washing as before. Next, 100 µL of AP substrate solution (Bio-Rad, Sydney, Australia) diluted in NPP buffer was added to each well and the plate was incubated until spots were visible, then washed with water. Spots were counted using an AID ELISpot reader (Melbourne, Australia) with the software ELISpot Reader version 6.0.

### 2.8. Statistical Analysis

Data were analysed using GraphPad Prism^®^ version 7.00. Student’s *t*-test was used for comparing the statistical significance of differences between two sets of data and corrected for multiple comparisons using the Holm–Sidak method, and ANOVA was used to compare three or more groups. Significant differences were denoted as * *p* < 0.05, ** *p* < 0.01, *** *p* < 0.001, **** *p* < 0.0001.

## 3. Results

### 3.1. CD4^+^ T Cells Express CXCR3 in Response to Pulmonary Vaccination with rIAV

**Figure 1:**
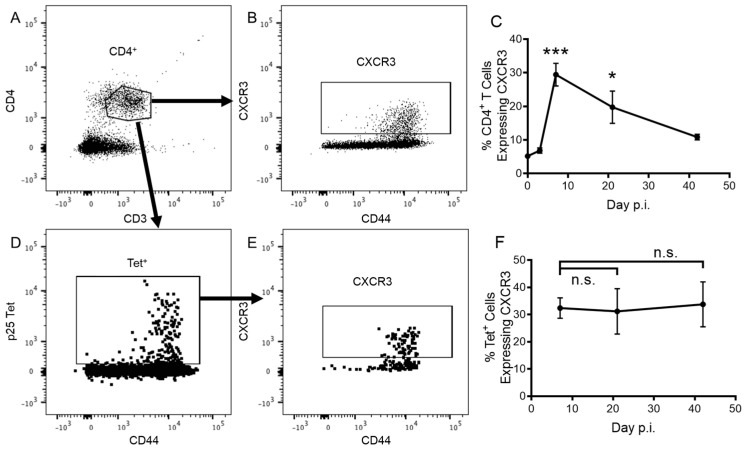
CXCR3-eGFP expression by pulmonary CD4^+^ T cells following PR8.p25 immunization. CiBER^+/−^ mice (n = 3–4) were immunized with 20 pfu PR8.p25 i.n. and pulmonary lymphocytes were isolated 3, 7, 21 and 42 days p.i. CXCR3-eGFP expression was measured by flow cytometry on lung CD4^+^ T cells (**A**,**B**) and p25-specific tet^+^ CD4^+^ T cells (**D**,**E**). Data are shown as the means ± SEM for the proportion of total CD4^+^ T cells (**C**) and p25-specific tet^+^ CD4^+^ T cells (**F**) expressing CXCR3-eGFP and are representative of two independent experiments. The significance of differences between the initial and later time points was determined by one-way ANOVA with multiple comparisons testing (* *p* < 0.05, *** *p* < 0.001).

To determine whether CXCR3 contributes to the cellular immune response to PR8.p25 vaccination, we immunized heterozygous CiBER mice, in which eGFP is expressed by 50% of CXCR3-expressing cells, with i/n PR8.p25, and analysed CXCR3 expression on CD3^+^ CD4^+^ T cells (Figure 1A,B) and p25-specific CD3^+^CD4^+^tet^+^ T cells (Figure 1C,D) by flow cytometry. In the lungs, the frequency of CD3^+^CD4^+^ T cells expressing CXCR3 peaked 7 days p.i. and declined until day 42, when CXCR3^+^CD4^+^ T cells remained more frequent than in unimmunized (0 days p.i.) mice (Figure 1E). CD3^+^CD8^+^ T cells also entered the lungs in response to rIAV PR8-p25 (Figure S3A) and showed a similar pattern of CXCR3 expression (Figure S3B). Vaccine-antigen-specific CD4^+^ T cells were identified with IA^b^-p25 MHC class II tetramers tagged with PE, which bind to p25-specific T cell receptors. These p25-tet^+^ CD4^+^ T cells were first detected after 7 days and a similar proportion of p25-tet^+^ CD4^+^ T cells expressed CXCR3 from 7 d.p.i. until 42 d.p.i. (Figure 1F).

**Figure 2:**
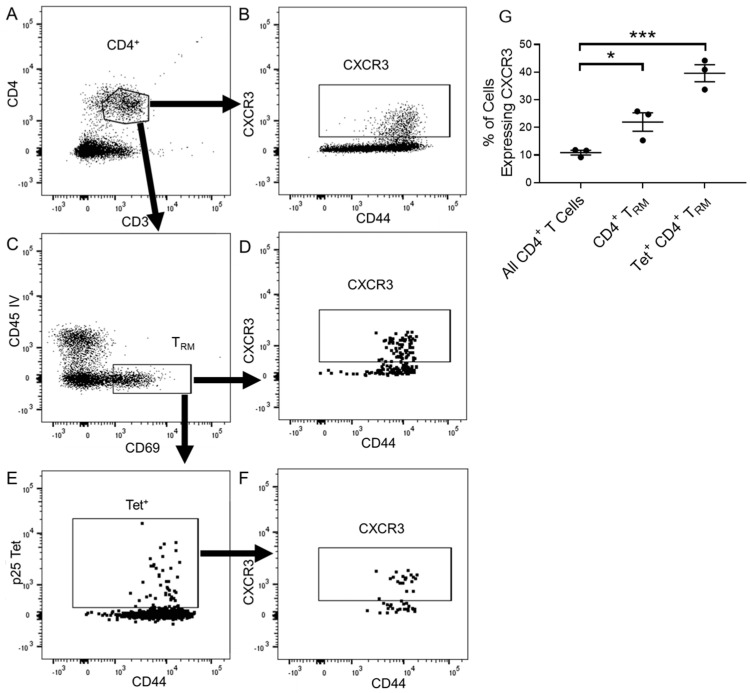
CXCR3-eGFP expression by T_RM_ after PR8.p25 immunization. CiBER^+/−^ mice (n = 3) were immunized with 20 pfu PR8.p25 i.n. and pulmonary lymphocytes were isolated 42 days p.i. following intravascular staining to identify lung-resident T cells. CXCR3-eGFP expression was examined on CD44^+^ CD4^+^ T cells (**A**,**B**), CD69^+^ IV^−^ CD44^+^ CD4^+^ T_RM_ (**C**,**D**), and p25-specific tet^+^ CD4^+^ T cell (**E**,**F**). CXCR3-eGFP expression on lung CD4^+^ T cells, CD4^+^ T_RM_ and CD4^+^ tet^+^ T_RM_ was then compared (**G**). Data are shown as the means ± SEM and are representative of two independent experiments. The significance of differences between the groups was determined by one-way ANOVA (* *p* < 0.05, *** *p* < 0.001).

We used intravascular staining (IVS) and CD69 expression to identify tissue-resident CD4^+^ T_RM_ (CD3^+^CD4^+^CD69^+^CD45IV^−^) in the lung parenchyma 6 weeks after PR8-p25 immunization (Figure 2A–F). A significantly higher proportion of parenchymal CD4^+^ T_RM_ expressed CXCR3 in comparison to total lung CD4^+^ T cells (Figure 2G), and 50% of p25-tet^+^ CD4^+^ T_RM_ were eGFP-positive in the heterozygous CiBER mice, indicating that nearly all antigen-specific CD4^+^ T_RM_ recruited to the lung expressed CXCR3 (Figure 2G).

### 3.2. CXCR3 Is Not Required for the Recruitment of CD4^+^ T Cells to the Lungs Following rIAV Vaccination

**Figure 3:**
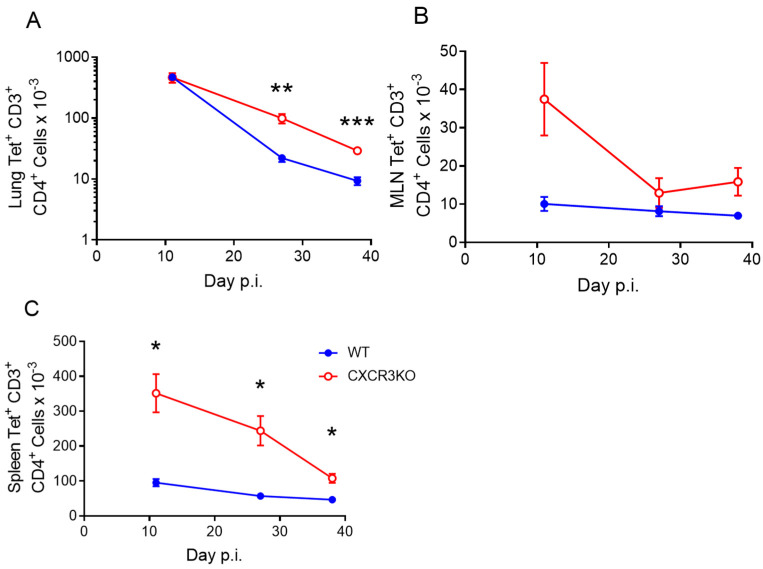
Increased numbers of p25 tetramer^+^ CD4^+^ T cells in CXCR3^−/−^ mice after PR8.p25 immunization. CXCR3^−/−^ and WT mice (n = 4–5) were immunized with 20 pfu PR8.p25 i.n. and cells were isolated from their lungs, MLNs and spleens at 11, 27 and 38 days post-immunization and analysed by flow cytometry. p25-specific tet^+^ CD3^+^ CD4^+^ T cells were measured in the lungs (**A**), MLNs (**B**) and spleens (**C**). Data are shown as the means ± SEM and are representative of two independent experiments. The significance of differences between the groups at each time point was determined by Student’s *t*-test (* *p* < 0.05, ** *p* < 0.01, *** *p* < 0.001).

To investigate whether CXCR3 is necessary for the induction of a p25-specific CD4^+^ T cell response to PR8.p25 or the formation of pulmonary CD4^+^ T_RM_, we immunized CXCR3^−/−^ and WT mice i/n with PR8.p25 and measured the CD4^+^ T cell response in the lungs, MLNs and spleens (Figure 3 and Figure S4A,C,E). At early time points, there were no significant differences in the total number of CD4^+^ T cells in the lungs or MLNs of CXCR3^−/−^ and WT mice, but a significant increase in the number of CD8^+^ T cells in the lungs and spleens of CXCR3^−/−^ mice compared to WT mice (Figure S4B,F). There was also a significant increase in the total number of CD4^+^ T cells in the spleens of CXCR3^−/−^ mice compared to WT mice (Figure S4E). We observed a trend towards greater numbers of CD4^+^ T cells in the lungs and MLNs of CXCR3^−/−^ mice at days 3 and 5 post-immunization, but this was abrogated at day 7 (Figure S4A,C). By day 11, we could detect vaccine-antigen-specific p25-tet^+^ CD4^+^ T cells. Equivalent numbers of these p25-specific cells were present in the lungs of CXCR3^−/−^ and WT mice at day 11 p.i. (Figure 3A). At later time points, however, there were significantly more p25-tet^+^ CD4^+^ T cells in the lungs, MLNs and spleens of CXCR3^−/−^ compared to WT mice (Figure 3A–C).

**Figure 4:**
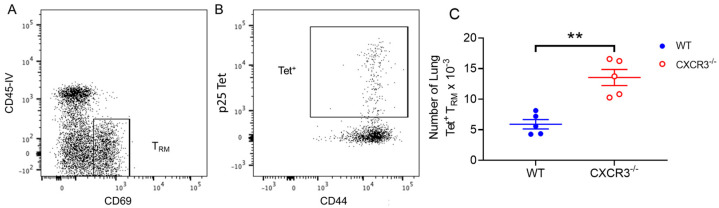
Increased numbers of p25-specific tetramer^+^ CD4^+^ T_RM_ in CXCR3^−/−^ mice after PR8.p25 immunization. CXCR3^−/−^ and WT mice (n = 5) were immunized with 20 pfu PR8.p25 i.n. and cells were isolated from their lungs 38 days post-immunization and analysed by flow cytometry. Following p25 gating on CD4^+^ T cells, T_RM_ and p25-specific specific tet^+^ CD3^+^ CD4^+^ T cells were identified by IVS (**A**) and tetramer staining (**B**), respectively, and tet^+^ T_RM_ were compared between groups (**C**). Data are shown as the means ± SEM and are representative of two independent experiments. The significance of differences between the groups was determined by Student’s *t*-test (** *p* < 0.01).

We then examined the impact of CXCR3 deficiency on retention of antigen-specific CD69^+^ CD44^+^ T_RM_ in the lung parenchyma following PR8.p25 immunization. Gating analysis was used to identify CD4^+^ T cells (Figure S1) that were CD45IV^−^CD69^+^ T_RM_ (Figure 4A) and p25-specific (Figure 4B). Strikingly, there were significantly more p25-tet^+^ CD4^+^ T_RM_ in the lungs of CXCR3^−/−^ than in WT mice (Figure 4C). Therefore, although CXCR3 is expressed on lung memory T cells following pulmonary immunization, the development and retention of lung-resident CD4^+^ T_RM_ is independent of CXCR3 expression.

To determine whether CXCR3^−/−^ mice had more severe PR8.p25 infections that contributed to their p25-specific CD4^+^ T cell responses, we monitored the weights of the infected CXCR3^−/−^ and WT mice. Both strains lost and regained similar amounts of weight, indicating their infections were equally severe (Figure S5). We also analysed viral loads in both strains by plaque assay at 3 and 7 days p.i and found no difference between their viral loads.

### 3.3. Pulmonary T Cell Cytokine Responses Are Independent of CXCR3 Following rIAV Vaccination

**Figure 5:**
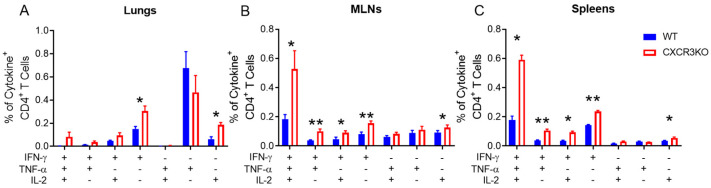
Cytokine expression in response to p25 in PR8.p25-immunized CXCR3^−/−^ mice. CXCR3^−/−^ and WT mice (n = 5) were immunized with 20 pfu PR8.p25 i.n. and cells were isolated from their lungs, MLNs and spleens 38 days post-immunization. They were stimulated with p25 overnight and analysed by flow cytometry. Boolean gating analysis was used to detect CD3^+^ CD4^+^ T cells that expressed IFN-γ, TNF-α and IL-2. Single- and multi-cytokine producing CD3^+^ CD4^+^ T cells were measured in the lungs (**A**), MLNs (**B**) and spleens (**C**). Data are shown as the means ± SEM and are representative of two independent experiments. The significance of differences between the groups was determined by Student’s *t*-test (* *p* < 0.05, ** *p* < 0.01).

To determine whether CXCR3 deficiency impaired the functional capacity of antigen-specific CD4^+^ T cells following PR8.p25 immunization, we measured the production of IFNγ, TNF and IL-2 following the ex vivo restimulation of lung, MLN and spleen cells from CXCR3^−/−^ and WT mice. We observed a trend towards increased percentages of pulmonary cytokine-producing CD4^+^ T cells from CXCR3^−/−^ mice, although only IFNγ^+^ and IL-2^+^ single-positive cell frequencies were significantly elevated in CXCR3^−/−^ mice compared to WT mice (Figure 5A). The MLNs and spleens of CXCR3^−/−^ mice showed increased frequencies of polyfunctional CD4^+^ T cells and all IFNγ-secreting CD4^+^ T cell subsets as well as IL-2^+^ single-positive CD4^+^ T cells (Figure 5B,C).

**Figure 6:**
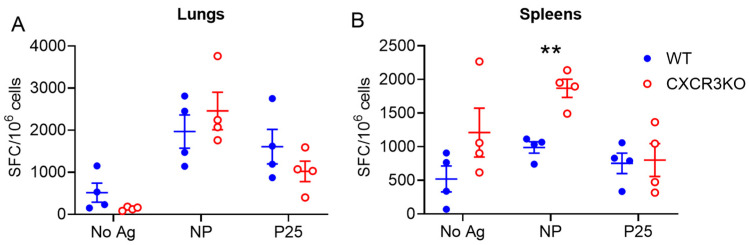
Lymphocytes from vaccinated CXCR3^−/−^ mice produce IFN-γ in response to CD4^+^ and CD8^+^ T cell vaccine antigens. ELISpot analysis of lung (**A**) and spleen (**B**) cells isolated from CXCR3^−/−^ and WT mice (n = 4) 38 days p.i. with PR8.p25. Cells were stimulated with either no antigen, H-2D^b^-restricted NP_366–374_ peptide recognised by CD8^+^ T cells, I-A^b^-restricted p25 recognized by CD4^+^ T cells, or concanavalin A. Data are shown as the means ± SEM and are representative of two independent experiments. The significance of differences between the groups was determined by Student’s *t*-test (** *p* < 0.01).

Additionally, we performed ELISpot analysis to compare T cell IFNγ responses to the NP_366–374_ and p25 epitopes of the PR8.p25 vaccine, which are recognised by CD8^+^ and CD4^+^ T cells, respectively. There were no differences between anti-NP and anti-p25 IFNγ secreting responses in the lungs (Figure 6A) or anti-p25 IFNγ responses in the spleens (Figure 6B). Significantly more splenocytes produced IFN-γ in response to NP in CXCR3^−/−^ mice than in WT mice (Figure 6B). These results demonstrate that the absence of CXCR3 does not impair T cell cytokine production.

### 3.4. CXCR3 Provides a Competitive Advantage to CD4^+^ T Cell Responses to rIAV Vaccination

**Figure 7:**
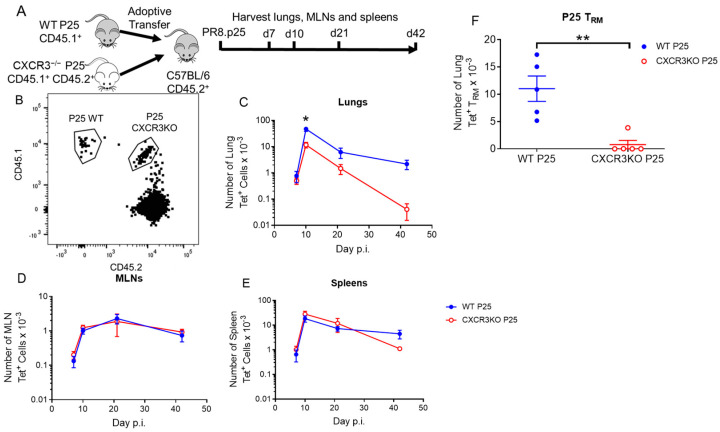
CXCR3^−/−^ p25-specific CD4^+^ T cells have a competitive disadvantage compared to WT p25-specific CD4^+^ T cells for recruitment to and retention in the lung. 25,000 CXCR3^−/−^ and WT P25 CD4^+^ T cells were transferred to C57Bl/6 mice (n = 4–6) 24 h prior to i.n. immunization with 20 pfu of PR8.p25 (**A**). After 7, 10, 21 and 42 days, lungs, MLNs and spleens were harvested, and cells were stained and analysed by flow cytometry. The congenic markers CD45.1 and CD45.2 were used to identify WT and CXCR3KO P25 cells (**B**). WT and CXCR3KO P25 cells were quantified in the lungs (**C**), MLNs (**D**) and spleens (**E**). WT and CXCR3KO P25 T_RM_ were quantified in the lungs at day 42. (**F**). Data are shown as the means ± SEM and are representative of two independent experiments. The significance of differences between the groups was determined by Student’s *t*-test (* *p* < 0.05, ** *p* < 0.01).

To determine whether CXCR3 confers a competitive advantage in the CD4^+^ T cell response to PR8.p25, we adoptively transferred 25,000 WT P25 cells and 25,000 CXCR3^−/−^ P25 cells into WT mice, immunized the mice with PR8.p25 i/n and measured the recruitment of antigen-specific T cells to the lungs via flow cytometry (Figure 7A,B). Both CXCR3^−/−^ and WT antigen-specific T cells were recruited to the lungs in similar numbers after 7 days (Figure 7C). From day 11 onwards, however, a smaller proportion of CXCR3^−/−^ than WT antigen-specific cells remained in the lungs, and these differences were significant at day 11 and day 42 (Figure 7C). There were no significant differences between CXCR3^−/−^ and WT antigen-specific T cells present at any time points in the MLNs or spleens (Figure 7D,E). Furthermore, there were significantly fewer p25 antigen-specific CXCR3^−/−^ CD45IV^−^ CD69^+^CD3^+^CD4^+^ T_RM_ in the lungs compared with WT P25 T cells at day 42 (Figure 7F). Therefore, although CXCR3 was not required for retention of antigen-specific T_RM_ in the lungs, the presence of this receptor provided a competitive advantage for the recruitment CD4^+^ T cells into the lung and their retention as antigen-specific T_RM._

## 4. Discussion

Vaccine-induced T_RM_ have recently been proposed as a correlate of protective TB immunity because of their rapid response to *M. tuberculosis* challenge at the site of infection and their role in long-term protection [9,21,39]. In human TB patients, T_RM_ accumulate at all infection sites, are highly activated, and elicit a rapid multifunctional cytokine response to restimulation with *M. tuberculosis* in vitro [40]. While the expression of CD69 and CD103 is generally accepted as identifying T_RM_, the need for additional well-validated and accurate T_RM_ markers [41,42] has prompted the evaluation of CD49a, CXCR6, CXCR3, CD101, PD-1, CD62L, KLRG1, and CX3CR1 in various studies [43]. This study focuses on CXCR3, which is highly expressed by T_RM_ and has been considered necessary for their development and retention [30,44,45]. The key findings of our study reveal that while the majority of vaccine-induced CD4^+^ T_RM_ in the lungs does express CXCR3 in response to pulmonary immunization, CXCR3 expression is not necessary for the formation and retention of CD4^+^ T_RM_ in the lungs.

CXCR3 plays a major role in immune responses to lung pathogens, being rapidly upregulated after T cell activation [46] and facilitating the migration of T cells to the lungs and draining lymph nodes [47]. CXCR3 ligands CXCL9, CXCL10 and CXCL11 are highly expressed in the lung in response to influenza [48] as well as *M. tuberculosis* [29,49] infection. Serum levels of CXCR3 ligands, in particular CXCL10 (or IP-10), have been studied as potential biomarkers of active TB disease [50] and the rapid response to TB treatment [51]. CXCR3 may also play a role in the induction of T cell memory as antigen recall responses from CD4^+^ and CD8^+^ T_CM_ and T_EM_ were largely restricted to CXCR3^+^ cell subsets [52,53]. In influenza infection, CXCR3 is important for promoting T follicular helper (T_FH)_ cell activity and antibody production [48], and in mouse TB models it is a marker for a protective antigen-specific CD4^+^ T cell subset [31]. In the non-human primate model of latent TB infection, there were high frequencies of CXCR3^+^CD4^+^ T cells in lung granulomas, inversely proportional to the *M. tuberculosis* bacterial load, and CXCR3 was co-expressed on the antigen-specific T cells producing IFNγ and IL-17 [54]. Paradoxically, the disruption of CXCR3-CXCL11 signalling in zebrafish increases resistance to mycobacterial infection [25], and CXCR3^−/−^ mice with a BALB/c background exhibited improved control of chronic *M. tuberculosis* infection [55]. This suggests that while CXCR3 expression may appear to correlate with protective immunity to TB infection, this relationship is not causal.

In this study CXCR3 was expressed by CD4^+^ T cells in the lungs following rIAV immunization with PR8.p25 (Figure 1E), and maintained on rIAV-induced, p25-specific CD4^+^ T cells for at least six weeks post-immunization (Figure 1F). This is consistent with previous findings showing that mucosal, but not peripheral, TB vaccination results in the generation of antigen-specific CXCR3^+^CD4^+^ T cells in the lung parenchyma [56]. The mice used in the current study were heterozygous CiBER females expressing the CXCR3 gene on both X chromosomes, but with eGFP under the control of the CXCR3 promoter on one chromosome. Because the Lyon effect results in gene expression in individual cells from only one X chromosome at random [57], only half of the T cells in the CiBER^eGFP/WT^ mice express the eGFP reporter gene when CXCR3 is expressed. Unlike surface staining analysis, which only detects CXCR3 molecules present on the cell surface, this model allows for the detection of all cells translating CXCR3-coding mRNA. Approximately 40% of p25-tetramer^+^ CD4^+^ T cells maintained eGFP expression until 42 d.p.i (Figure 1F), indicating that 80% of p25-specific cells expressed CXCR3 for at least six weeks following vaccination. As CXCR3 is believed to be required for T cell migration into the lung [30] and CXCR3 deficiency has been reported to decrease the rate of CD4^+^ T cell entry to the lung parenchyma by from five- to ten-fold [27], we examined the impact of deleting CXCR3 expression in this model of mucosal immunization.

Unexpectedly, CXCR3^−/−^ mice had equivalent numbers of p25-specific CD4^+^ T cells to WT mice in the lungs 11 days after PR8.p25 immunization, and increased numbers of p25-specific CD4^+^ T cells at days 28 and 38 p.i. (Figure 3A). CXCR3 is upregulated upon activation of naïve T cells and remains highly expressed on Th1 CD4^+^ T cells [58,59]. In addition, interactions between CXCR3 and its ligands facilitate the movement of CD4^+^ Th1 cells to sites of inflammation [60]. However, our results indicate that the deletion of CXCR3 resulted in enhanced antigen-specific CD4^+^ T cell responses to rIAV immunization in the lungs, suggesting that the presence of CXCR3 signalling is not wholly beneficial in the context of mucosal TB vaccination. This is not true for vaccine-induced antigen-specific CD8^+^ T cells during *M. tuberculosis* infection in mice, which exhibit impaired entry to the lung parenchyma when treated with a CXCR3-blocking antibody [30]. Notably, we showed that CXCR3 expression was not necessary for the formation of CD4^+^ T_RM_, and the CXCR3^−/−^ mice generated more p25-specific CD4^+^ T_RM_ than WT mice (Figure 4C). CXCR3^−/−^ mice also had more p25-specific CD4^+^ T cells in their spleens than WT mice at all studied time points (Figure 3C), indicating they had a larger systemic memory CD4^+^ T cell response in addition to their increased number of T_RM_. One possible explanation for these differences is that, in CXCR3^−/−^ mice, CD4^+^ T cells employ compensatory mechanisms for lung-homing that enhance memory cell formation through altered tissue-homing or enhanced proliferation or survival. This has implications for future vaccine strategies, as techniques to suppress CXCR3 signalling using drugs or vaccines that prevent CXCR3 ligand expression may be employed with future vaccines to induce a larger systemic or resident memory CD4^+^ T cell response. This approach proved effective for CD8^+^ T cell responses in mice [61], where the temporary pharmaceutical inhibition of CXCR3 and CCR5 resulted in enhanced CD8^+^ T cell responses. When CXCR3^−/−^ p25-specific CD4^+^ T cells were adoptively transferred into WT hosts along with WT p25-specific T cells, however, the CXCR3-deficient CD4^+^ T cells entered the lungs in reduced numbers compared to their WT counterparts at early and later time points p.i. (Figure 7C), and there was a marked reduction in the number of antigen-specific T_RM_ in the lungs (Figure 7F). Furthermore, CXCR3^−/−^ and WT P25 cells remained in equal numbers in the MLNs and spleens at all time points (Figure 7D,E), in contrast with CXCR3^−/−^ mice, which had increased numbers of splenic p25-specific CD4^+^ T cells (Figure 3C). This shows that CXCR3^−/−^ CD4^+^ T cells compete equally with their WT counterparts in differentiating to circulating memory phenotypes, but not in lung T_RM_ formation. This may be because WT CD4^+^ T cells outcompete CXCR3^−/−^ T cells in accessing cytokines or physical spaces needed for differentiation into CD4^+^ T_RM_. This contrasts with the study by Dhume et al. [62], who adoptively transferred T-bet-deficient antigen-specific CD4^+^ T cells with decreased CXCR3 expression, along with WT cells into mice infected with influenza A virus. They showed that T-bet-deficient cells entered the lungs in reduced numbers, similarly to the CXCR3^−/−^ cells examined here. However, they first saw defective lung entry after 7 days, when we observed no differences. They also noted enhanced splenic memory cell formation by T-bet^−/−^ CD4^+^ T cells, which did not occur with adoptively transferred CXCR3^−/−^ CD4^+^ T cells. These results expand upon observations that CXCR3^+^ CD4^+^ T cells purified from the lungs of *M. tuberculosis*-challenged mice will migrate back into the lungs [63,64], but the clinical impact of this competitive advantage of WT over CXCR3^−/−^ p25-specific CD4^+^ T cells remains to be determined.

CXCR3 plays a role in T cell migration but does not affect antigen recall responses in T cells. Lymphocytes from both CXCR3^−/−^ and WT mice vaccinated with PR8.p25 expressed IFN-γ in response to CD4^+^ and CD8^+^ T cell vaccine epitopes after 6 weeks (Figure 5). In addition, CXCR3^−/−^ mice displayed increased polyfunctional cytokine CD4^+^ T cell responses following p25 stimulation compared to WT mice. Such polyfunctional T cell responses are associated with protection against *M. tuberculosis* infection in BCG-vaccinated mice [65]; however, they do not correlate with protective immunity against TB in humans [7,66]. Furthermore, this study demonstrates that the number and functionality of antigen-specific CD4^+^ T cells in the lungs and the retention of antigen-specific T_RM_ following mucosal TB immunization is independent of the chemokine receptor, CXCR3. In fact, memory CD4^+^ T cell responses to the pulmonary vaccine were increased in the absence of CXCR3. This finding is consistent with the observation that CXCR3-deficient BALB/c mice infected by aerosol with *M. tuberculosis* developed increased memory T cell responses that were associated with increased clearance of the mycobacteria at 12 and 24 weeks [55]. The mechanism by which CXCR3 regulates memory CD4^+^ T cell formation in response to rIAV vaccination or *M. tuberculosis* infection is unknown. CXCR3 expression did not affect CD4^+^ T cell entry into the lungs up to 11 days after immunization, but CXCR3^−/−^ mice developed increased p25-specific splenic CD4^+^ T cells at days 11 and 28, indicating that these underwent increased expansion or decreased contraction prior to this. This could be related to impaired T cell-DC interactions in lymph nodes, as has been reported previously [32]. To elucidate this mechanism, future studies could employ imaging techniques to assess CD4^+^ T cell localisation with antigen in both the lymph nodes and lung, as well as the impact of CXCR3 deletion on early T cell expansion and differentiation.

In conclusion, while CXCR3 provides an advantage to CD4^+^ T cells for recruitment to the lungs in response to this pulmonary TB vaccine, it is not essential for the development and retention of lung-resident memory CD4^+^ T cells. This has implications for designing vaccines that aim to induce increased memory CD4^+^ T cell responses, including T_RM_, in the lungs at the site of pathogen exposure.

## Figures and Tables

**Table 1:** Monoclonal antibodies and staining reagents used in experiments.

Assay	Marker	Fluorophore	Clone	Manufacturer
Flow Cytometry	CD3	PerCP-Cy5.5	145-2C11	BioLegend (San Diego, CA, USA)
	CD3	PE-Cy7	145-2C11	BD Biosciences (Sydney, Australia)
	CD3	N/A	145-2C11	BD Biosciences
	CD28	N/A	37.51	BD Biosciences
	CD4	AF700	RM4-5	BD Biosciences
	CD44	FITC	IM7	BD Biosciences
	CD45.1	Biotin	A20	BD Biosciences
	CD45.2	PerCP-Cy5.5	104	BioLegend
	CD62L	eF450	MEL-14	eBioscience (San Diego, CA, USA)
	CD69	PE	H1.2F3	BD Biosciences
	IFN-γ	PE	XMG1.2	BD Biosciences
	IFN-γ	FITC	XMG1.2	BD Biosciences
	TNF	APC	MP6-XT22	BD Biosciences
	TNF	PE	MP6-XT22	BioLegend
	IL-2	APC	JES6-5H4	BioLegend
	UV LIVE/DEAD^®^	UV LIVE/DEAD^®^	N/A	BioLegend
	Biotin	Pacific orange	N/A	Invitrogen (Waltham, MA, USA)
	p25 tetramer	APC	N/A	NIH Tetramer Core Facility
ELISpot	IFN-γ	N/A	AN18	Produced in house
	IFN-γ	N/A	XMG1.2	Produced in house

## Data Availability

The data supporting the reported results are available from the authors.

## Supplementary Materials

The following supporting information can be downloaded at: https://www.mdpi.com/article/10.3390/vaccines11101549/s1, Figure S1: Gating strategy for flow cytometry analysis; Figure S2: Gating strategy for flow cytometry ICS analysis; Figure S3: CXCR3-eGFP expression by pulmonary CD8^+^ T cells following PR8.p25 immunization; Figure S4: Early lymphocyte response of CXCR3^−/−^ mice to PR8.p25 immunization; Figure S5: CXCR3^−/−^ mice show similar systemic responses to PR8.p25 infection.
