# Supplementary material for: CXCR3 Provides a Competitive Advantage for Retention of Mycobacterium tuberculosis-Specific Tissue-Resident Memory T Cells Following a Mucosal Tuberculosis Vaccine
Source: Vaccines (Basel). 2023 Sep 29;11(10):1549. doi: 10.3390/vaccines11101549 (PMC10611282; doi:10.3390/vaccines11101549)
Supplement: Supplementary file 1 [file vaccines-11-01549-s001.zip › vaccines-2622581-supplementary.pdf]

Armitage E., et al, CXCR3 provides competitive advantage for retention of *M. tuberculosis*-specific tissue resident memory T cells following a mucosal tuberculosis vaccine.

Supplementary Material

1 Supplementary Figures and Tables

1.1 Supplementary Figures

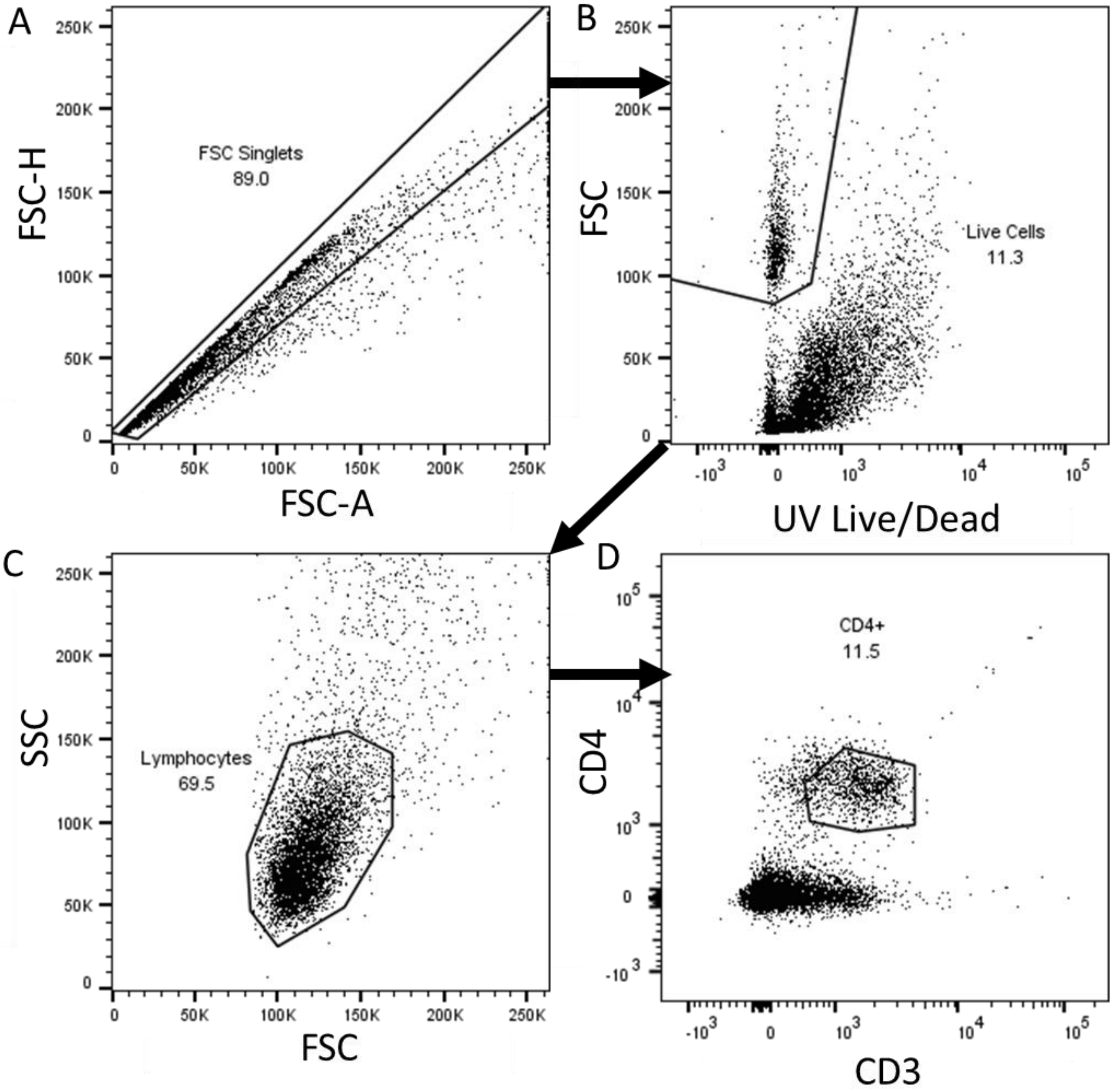

Figure S1. Gating strategy for flow cytometry analysis. (A) Exclusion of doublets based on differences between forward scatter area and height. (B) Exclusion of dead cells and debris using UV Live/Dead staining. (C) Gating of lymphocytes. (D) Gating of $CD3^{+}$ $CD4^{+}$ T cells.

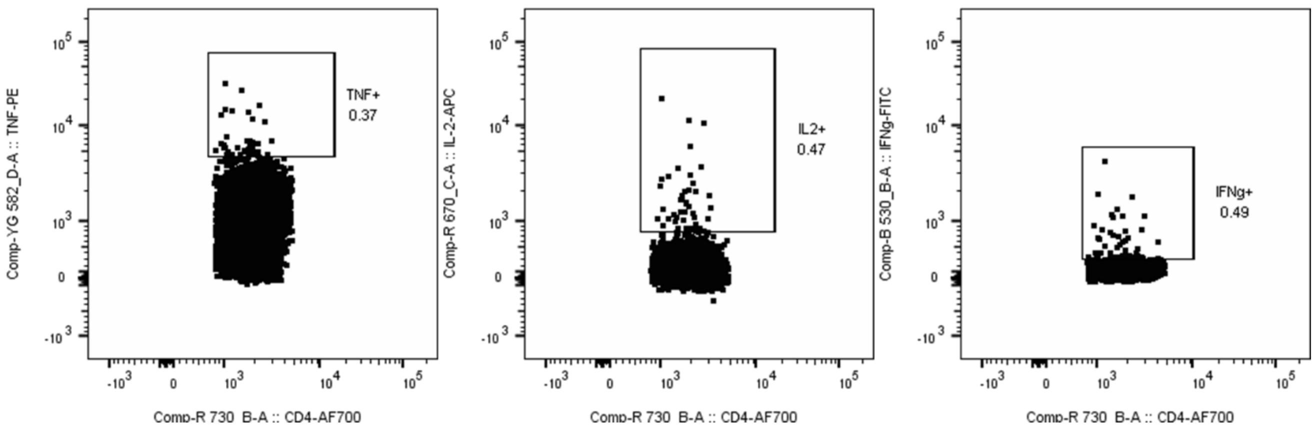

Figure S2. Gating strategy for flow cytometry ICS analysis. CD3$^+$ CD4$^+$ T lymphocytes that were stained by ICS to identify cells that expressed IFN-γ (A), TNF-α (B) and IL-2 (C). Boolean gating was used to identify cells that expressed multiple or single cytokines.

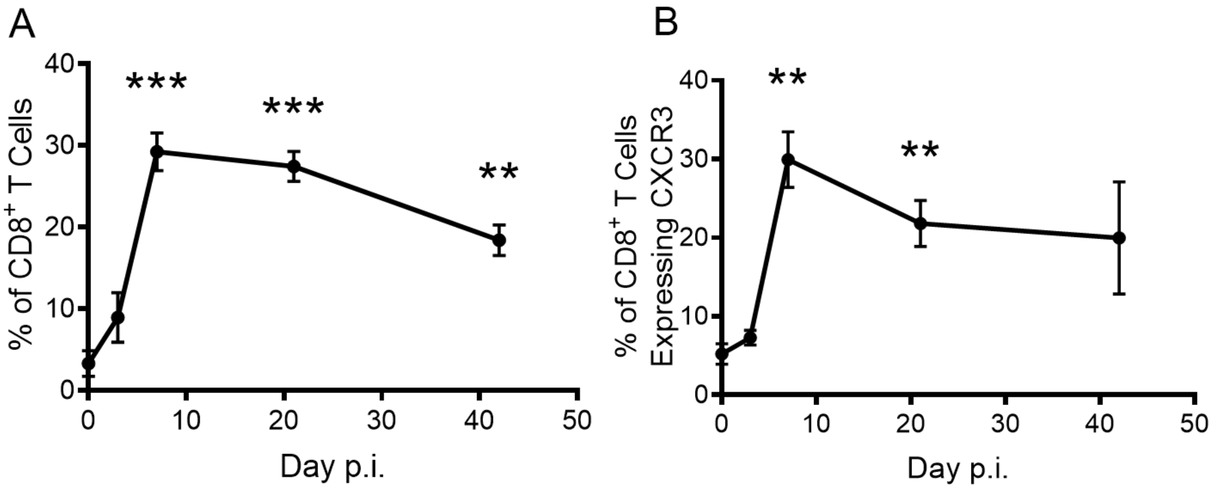

Figure S3. CXCR3-eGFP expression by pulmonary CD8$^+$ T cells following PR8.p25 immunization. CiBER$^{+/-}$ mice (n = 3-4) were immunized with 20 pfu PR8.p25 i.n. and pulmonary lymphocytes were isolated 3, 7, 21 or 42 days post-immunization. CD8$^+$ T cells (A) and CXCR3$^+$ CD8$^+$ T cells (B) were quantified following immunization. Data are shown as the means ± SEM. The significance of differences between the initial and later time points was determined by one-way ANOVA with multiple comparisons testing (**P<0.01, ***P<0.001).

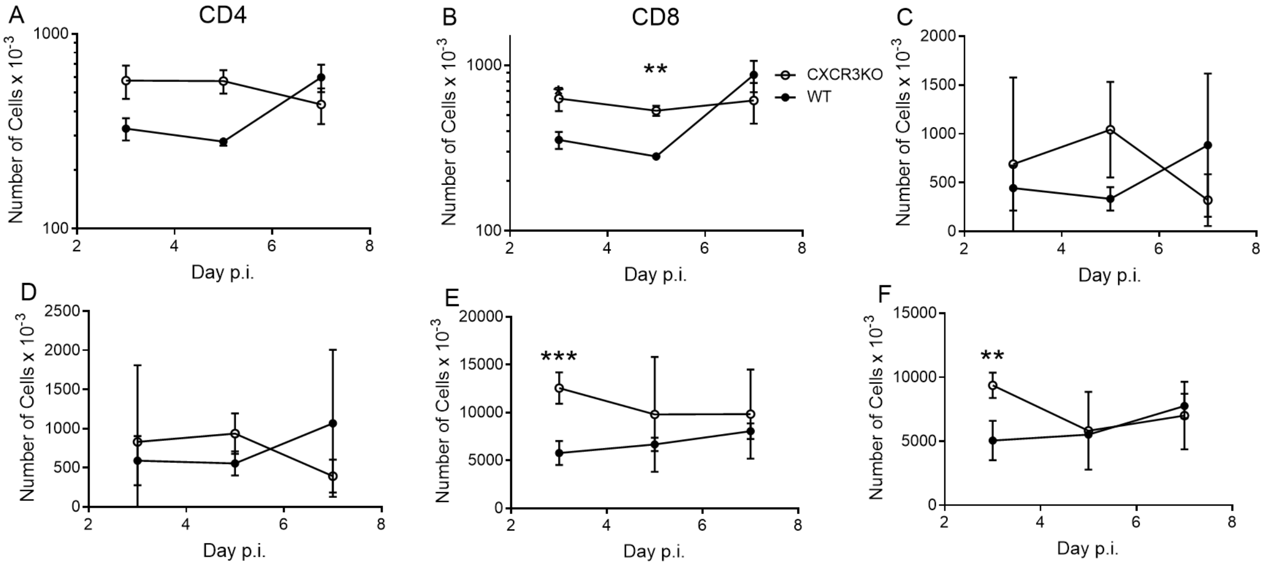

Figure S4. Early lymphocyte response of CXCR3$^{-/-}$ mice to PR8.p25 immunization. CXCR3$^{-/-}$ (open circle) and WT (closed circle) mice (n = 4) were immunized with PR8.p25 (20 pfu) and lymphocytes were isolated from their lungs, MLNs and spleens at 3, 5 and 7 days post-immunization and analyzed by flow cytometry. CD4^{+} and CD8^{+} T cells were measured in their lungs (A-B), MLNs (C-D) and spleens (E-F). Data are shown as the means ± SEM. The significance of differences between the groups was determined by Student's t-test (**P<0.01, ***P<0.001).

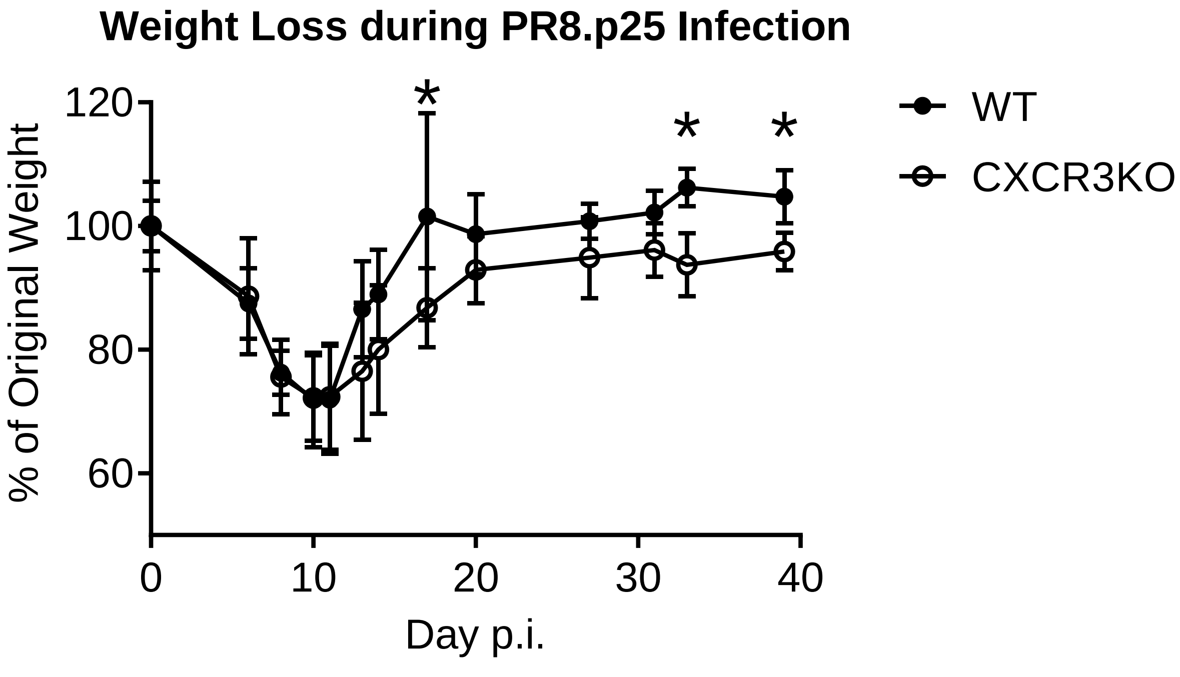

Figure S5. CXCR3$^{-/-}$ mice show similar systemic responses to PR8.p25 infection. CXCR3$^{-/-}$ and C57BL/6 WT mice were immunized with 20 pfu PR8.p25 i.n. and weighed following infection. Data are shown as the mean % of original weight ± SEM. The significance of differences between the groups was determined by Student's t-test (*P<0.05).
